# From CAR-T Cells to CAR-NK Cells: A Developing Immunotherapy Method for Hematological Malignancies

**DOI:** 10.3389/fonc.2021.720501

**Published:** 2021-08-06

**Authors:** Hui Lu, Xiaoyan Zhao, Ziying Li, Yu Hu, Huafang Wang

**Affiliations:** Department of Hematology, Union Hospital, Tongji Medical College, Huazhong University of Science and Technology, Wuhan, China

**Keywords:** chimeric antigen receptor, natural killer cells, T cells, immunotherapy, hematological malignancies

## Abstract

The approval of CD19 chimeric antigen receptor (CAR)-engineered T (CAR-T) cell products in B-cell malignancies represents a breakthrough in CAR-T cell immunotherapy. However, the remaining limitations concerning the graft-versus-host disease (GVHD) and other adverse effects (e.g., cytokine release syndromes [CRS] and neurotoxicity) still restrict their wider applications. Natural killer (NK) cells have been identified as promising candidates for CAR-based cellular immunotherapy because of their unique characteristics. No HLA-matching restriction and abundant sources make CAR-engineered NK (CAR-NK) cells potentially available to be off-the-shelf products that could be readily available for immediate clinical use. Therefore, researchers have gradually shifted their focus from CAR-T cells to CAR-NK cells in hematological malignancies. This review discusses the current status and applications of CAR-NK cells in hematological malignancies, as well as the unique advantages of CAR-NK cells compared with CAR-T cells. It also discusses challenges and prospects regarding clinical applications of CAR-NK cells.

## Introduction

Hematological malignancies, which contribute to approximately 7% of all newly diagnosed cancers, represent a class of malignant clonal diseases originating in the hematopoietic system, mainly including leukemia, lymphoma and multiple myeloma (MM) (1, 2). Allogeneic hematopoietic stem cell transplantation (allo-HSCT), considered “the ancestor of immunotherapy” in hematological malignancies, is often regarded as the sole curative approach for hematological malignancies (3, 4). Although the overall survival of hematological malignancies has been improved due to improvements in allo-HSCT, the relapse of underlying malignancy remains a major cause of failure or death after transplantation (5).

Immunotherapy (e.g., cytokine therapy, immune checkpoint blockade, and CAR-T cell therapy) has enriched treatment methods and improved the prognosis of patients with hematological tumors (3, 6). CAR-T cell therapy has stood the test of time for over 26 years (7). The approvement of four CD19 CAR-T cell products, tisagenlecleucel (tisa-cel), axicabtagene ciloleucel (axi-cel), brexucabtagene autoleucel, and lisocabtagene maraleucel, is a breakthrough for the use of engineered T cells in hematological malignant tumors (8–10). By carrying CARs, CAR-T cells can directly target specific tumor antigens, while enhancing the targeted toxicity and killing effects. Various types of CAR-T cells have been used for treating hematological malignancies (11). Despite considerable progress in CAR-T cell-based immunotherapy for patients with blood diseases, there remain restrictions that inhibit its broader application in future treatment of hematological malignancies: (1) CRS and neurotoxicity are notable acute side effects that occur during CD19 CAR-T cell therapy (12); (2) on-target off-tumor effects may occur related to the recognition of molecular biomarkers that are also expressed on healthy tissues (e.g., B cell aplasia in anti-CD19/CD20 CAR-T cell therapy) (13); (3) antigen escape/loss may lead to disease relapse (e.g., typical CD19-negative relapse in B-cell malignancy) (14); (4) life-threatening GVHD may be caused by allogeneic CAR-T cells (15); (5) restrictions concerning inclusion in commercialized and “off-the-shelf” products have been implemented because of the HLA restriction and insufficient sources of T cells. Currently, various strategies are under investigation to overcome these obstacles; additionally, researchers are seeking alternative immune effector cells that can be modified with CARs and then used in immunotherapy.

Recently, with the increasing attention toward the unique characteristics and specialized cytotoxicity of NK cells, the research focus has shifted from T cells to NK cells. NK cells, which belong to the innate lymphoid cell family, are a class of cytotoxic immune cells that have been functionally identified by their “natural” ability (16, 17). Human NK cells are characterized by a CD3^-^CD56^+^ immunophenotype and could be subdivided into two subgroups: CD56^bright^CD16^low/-^ cells (a less mature population) and CD56^dim^CD16^bright^ cells (a mature population of highly cytotoxic cells) (18). Unlike T lymphocytes, NK cells serve to target tumors without pre-sensitization or HLA-matching. Additionally, clinical evidence shows that adoptive transfusion of allogeneic NK cells rarely causes GVHD (19–21), moreover, NK cells may even protect from GVHD by targeting the recipient’s dendritic cells (22). Various approaches are used for NK cell-based immunotherapy. First, the addition of cytokines can enhance the activation, proliferation, and persistence of NK cells both *in vivo* and *in vitro* (23). Second, various antibodies have been investigated to further boost the killing activities of NK cells through multiple mechanisms: (1) monoclonal antibodies (mAbs), which target specific tumor-associated antigens (TAAs), have been approved for the treatment of hematological malignancies based on NK cell-mediated antibody-dependent cell-mediated cytotoxicity (ADCC) (24); (2) CD16 bi- and tri- killer cell engagers (CD16 BiKEs and CD16 TriKEs) are novel antibodies that can simultaneously bind two or three separate and unique antigens to strongly activate NK cell function, one is NK cell activating receptor CD16 and the other one or two are TAAs (25); (3) mAbs that target immune checkpoints or their corresponding ligands could restore the anti-tumor function of NK cells (26, 27); (4) mAbs targeting NK cell inhibitory receptors (e.g., KIRs and NKG2A) also remain under investigation (28, 29). Third, transfusion of NK cells is an effective adoptive immunotherapy method to improve the number and the function of NK cells (30). These approaches used for NK cell therapy are applicable for CAR-NK cell therapy.

Because of their greater safety, enhanced feasibility, and superior cytotoxicity, NK cells have been selected as a novel candidate platform for CAR-engineering. Compared with CAR-T cell therapy, there has been no evidence of CRS, neurotoxicity, or GVHD when using CAR-NK therapy for hematological malignancies (31). Notably, the feasibility of various sources of NK cells may enable CAR-NK cells to be included in “off-the-shelf” products for immediate clinical use. Some clinical trials regarding solid tumors and hematological malignancies have shown the impressive efficacies of CAR-NK cells (31, 32). In this review, we discuss the structure of CAR-NK cells, the current status and applications of CAR-NK cells in hematological malignancies, and the unique advantages of CAR-NK cells compared with CAR-T cells. We also discuss challenges and prospects regarding clinical applications of CAR-NK cells.

## CAR-NK Cell Engineering

Similar to the structures of CAR-T cells, CAR-NK cells are composed of CARs (genetically engineered transmembrane receptors) and effector cells (NK cells).

### CAR Constructs

**Figure 1:**
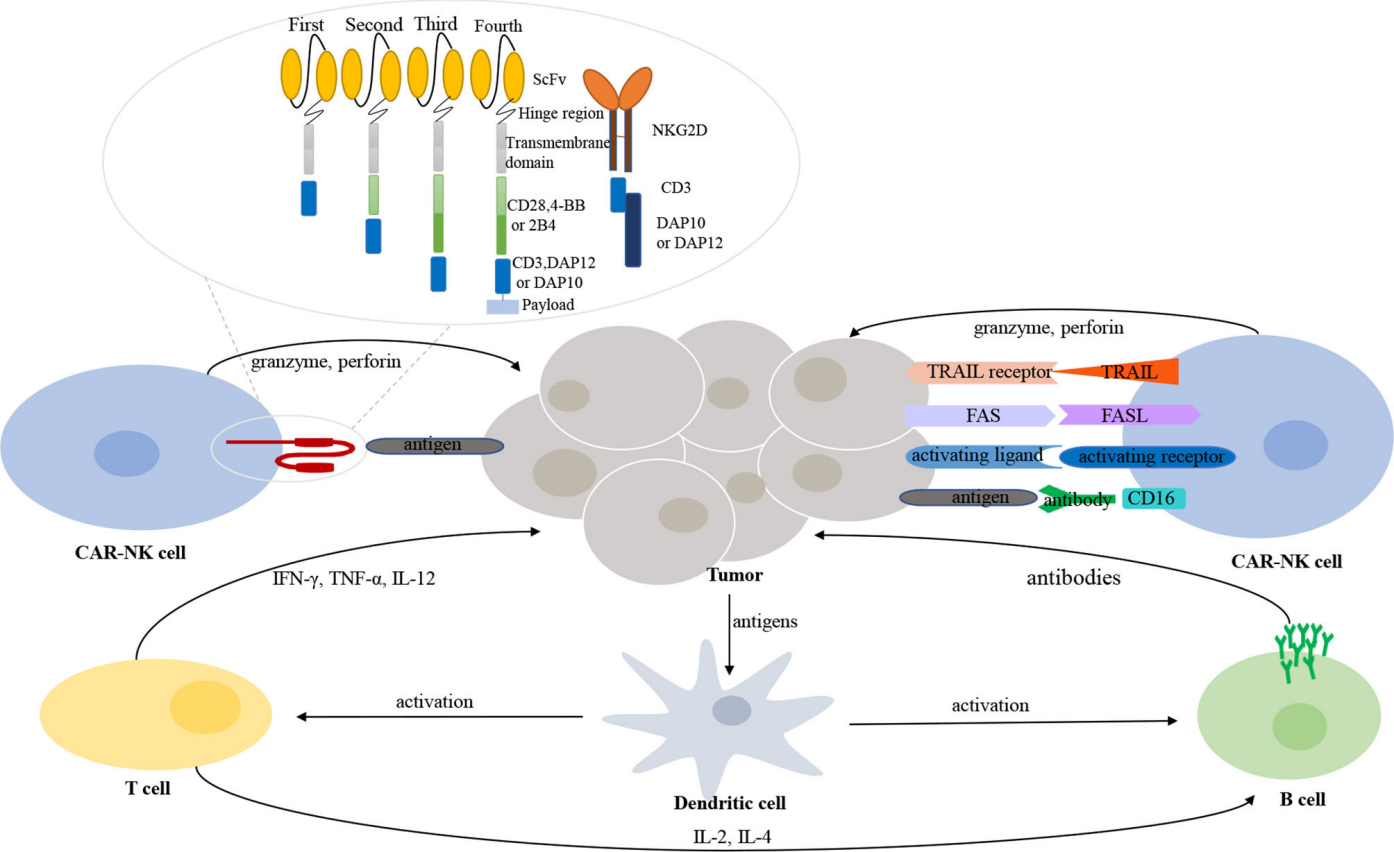
The interaction between immune cells and tumor cells in tumor microenvironment. CAR-NK cells recognize and exert their killing effects through both CAR-dependent and CAR-independent manners. Different generations and types of CAR-NK cells. A CAR structure contains an extracellular binding domain (e.g., ScFv or NKG2D), a hinge region, a transmembrane domain, and one or more intracellular signaling domains.

The CAR construct is critical for activating CAR-transduced cells. CARs used in CAR-NK cells are generally similar to those used in CAR-T cells. A CAR always comprises four components: an extracellular binding domain, a hinge region, a transmembrane domain, and one or more intracellular signaling domains (**Figure 1**). The extracellular binding domain confers specificity to CAR-modified effector cells by means of targeting TAAs. The hinge region connects the extracellular binding domain with the transmembrane domain. The intracellular signaling domains, which determine the strength of the activation signal and affect the killing activity, are of different compositions in various generations of CARs. The first generation of CARs typically contained only the CD3ζ activation signaling domain, while the second and third generations of CARs combined one or two additional costimulatory molecules (e.g., CD28, ICOS, 4-1BB, CD27, OX40, and CD40). Among these molecules, CD28 and 4-1BB are the most commonly used (33, 34). The fourth generation of CARs, designed by Chmielewski and colleagues, endow the effector immune cells with two transgenic products, the CAR and the transgenic payload. The fourth generation of CARs is focused on removing the current limitations of CAR-based cellular therapy and thus further improving the functions of effector cells (35).

In addition to the common CARs that are applicable for both CAR-T cells and CAR-NK cells, researchers have exploited other molecules that could serve as the activation signaling domain and be more suitable for NK cells. CD244(2B4), a member of the signaling lymphocyte activation molecule (SLAM) family, could also be used as a costimulatory molecule. Increased capacity of signal enhancement and augmented natural cytotoxicity against tumor cells are induced by the upregulation of 2B4 in NK cells (36). DNAX-activation protein (DAP) 12, expressed on NK cells, participates in signal transduction involving NK activating receptors natural-killer group 2 (NKG2) member C (NKG2C) and NKp44; DAP10 also participates in signal transduction involving NKG2D (37, 38). Therefore, DAP12 and DAP10 can transduce intracellular signals in CAR-NK cells. Moreover, NK cells engineered with DAP12-based CARs performed better than NK cells engineered with CD3ζ-based CARs (37). Cumulative evidence demonstrates that NKG2D ligands are overexpressed in several hematological malignancies. Therefore, the CAR construct, NKG2D-DAP10-CD3ζ, which targets the NKG2D ligands, is of great potential in blood cancers. The safety and enhanced killing activity of NKG2D-DAP12-CD3ζ CAR-NK cells for colorectal cancer have been proven in a clinical trial (39).

### NK Cells

**Figure 2:**
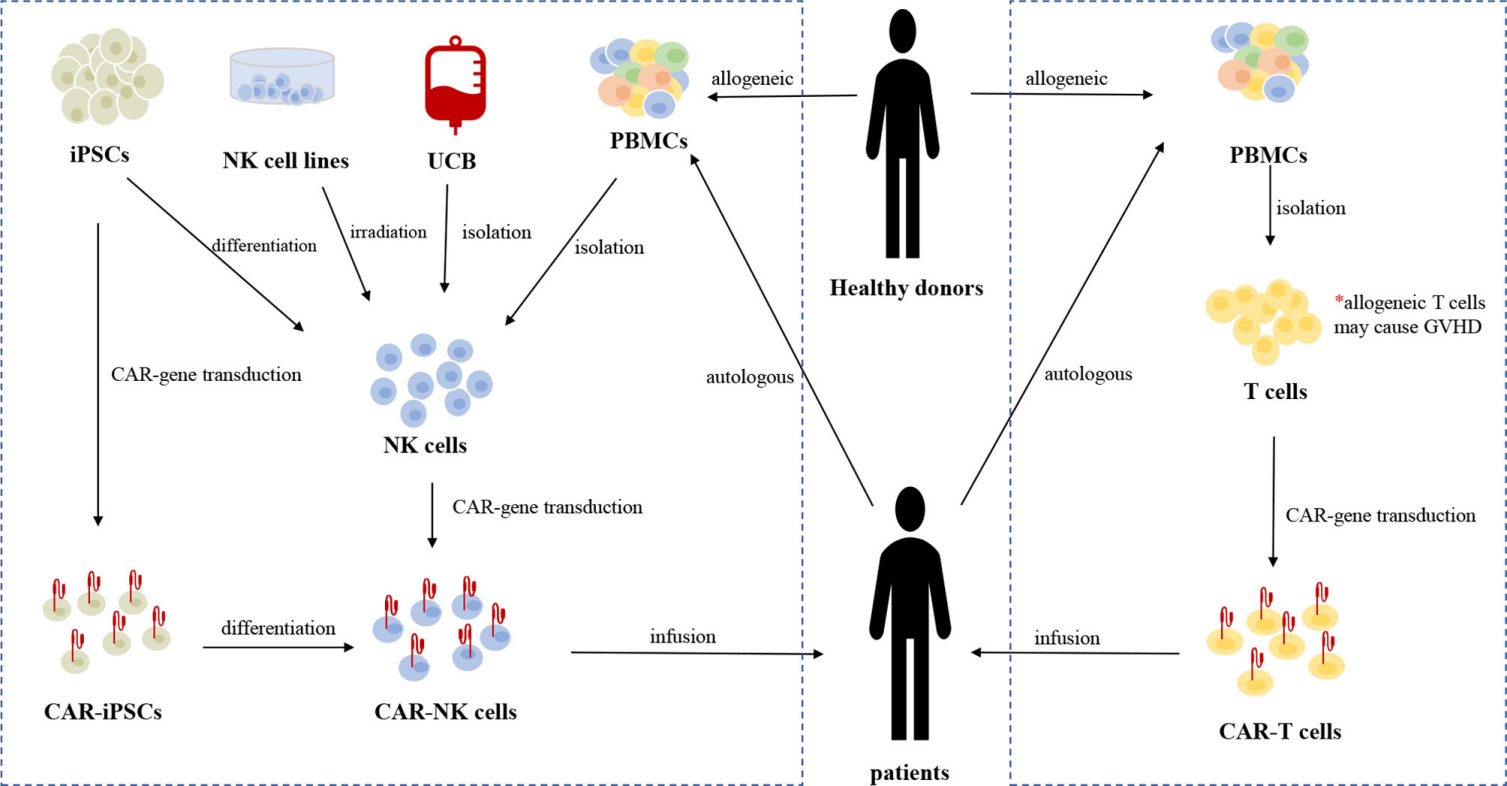
The sources and manufacturing process of CAR-NK cells and CAR-T cells, respectively. Current sources of clinical-grade NK cells include the NK92 cell line, autologous or allogeneic PBMC-derived NK cells, UCB-derived NK cells, and iPSCs-derived NK cells. iPSCs can be genetically engineered with CAR-gene and the resulting CAR-iPSCs can be differentiated into homogeneous CAR-NK cells for clinical use. Irradiation is necessary before the infusion of CAR-NK92 cells to eliminate the potential risk of malignant transformation. At present, autologous PBMC-derived T cells are the main source of T cells for CAR-gene engineering. Allogeneic T cells from healthy donors may cause life-threatening GVHD due to HLA restriction.

Various sources of allogeneic NK cells are available for producing CAR-NK cells (**Figure 2**). Current sources of clinical-grade NK cells include the NK92 cell line, peripheral blood mononuclear cells (PBMCs), umbilical cord blood (UCB), and induced pluripotent stem cells (iPSCs). Human embryonic stem cells (hESCs) and CD34+ hematopoietic progenitor cells (HPCs), also serve as sources of NK cells (40, 41).

The NK92 cell line is commonly used in adoptive immunotherapy. NK92 cells can be easily and reproducibly expanded from a good manufacturing practice (GMP)-compliant cryopreserved master cell bank (42). There are no killer immunoglobulin-like receptors (KIRs) or CD16 expressed on NK92 cells; thus, they cannot mediate ADCC (43). Although NK92 cells exhibit a poorly cytotoxic immunophenotype (CD56^bright^CD16^low/-^), the NK92 cell line is a highly cytotoxic cell line that expresses high levels of cytolytic pathway molecules (e.g., perforin and granzyme B) (44). Irradiation is necessary before infusion because of their chromosomal abnormalities and risk of malignant transformation (45). A novel type of CAR-NK cell line, CAR-KHYG-1, has shown antitumor effects against glioblastoma cells, suggesting that KHYG-1 may be a new option for CAR-NK cell therapy (46).

Approximately 90% of NK cells isolated from PBMCs are CD56^dim^CD16^bright^ cells, which typically represent a mature population with increased cytotoxicity and reduced proliferative capacity (47). There is no need for irradiation before infusion of PBMC-derived CAR-NK cells because of their lower risk. A great number of NK cells for GMP-grade clinical application can be obtained after isolation, purification, stimulation, and expansion of PBMCs derived from healthy donors or directly from patients themselves (48). However, similar to what happens with autologous T cells, obtaining autologous NK cells may be difficult in patients who have been heavily pre-treated with chemotherapy. Thus, there is a need to discover additional sources of allogeneic NK cells (rather than cells only from healthy donors) as platforms for CAR engineering. Furthermore, the purification is a vital procedure when obtaining allogeneic PBMC-derived NK cells, because residual B cells may cause lymphoma (due to reactivation of Epstein–Barr virus) and residual T cells may cause GVHD (49).

NK cells comprise approximately 30% of lymphocytes in UCB and 10% in the corresponding PB (50, 51). The capacities for cryopreservation and easy collection make UCB-derived NK cells appropriate for off-the-shelf applications (52). There are more than 600,000 UCB units stored in UCB banks, which allows the selection of donors with specific HLA types (53). There are few T cells in UCB and most are immature, thus minimizing the risk of GVHD (54, 55). A large proportion of UCB NK cells exhibit a naïve phenotype with decreased expression of adhesion molecules, CD16, KIRs, perforin, and granzyme B, as well as increased expression of inhibitory molecules (e.g., NKG2A), thus leading to lower cytotoxicity against tumor cells compared with their PB counterparts (52).

iPSCs, derived from non-hematopoietic cells, are another important stem-cell source of allogeneic NK cells. UCB and PBMCs generally cannot provide a homogeneous NK cell population and function in multiple-dosing strategies because it is unpractical to obtain cells from a single renewable source. However, only one single CAR-engineered iPSC cell is sufficient for differentiation into a large number of highly homogeneous CAR-NK cells for clinical use; therefore, repeated doses could be administered to patients to overcome the short NK cell lifespan (19, 52, 56, 57). Similar to NK92 cells and UCB-NK cells, iPSC-derived NK (iNK) cells exhibit an immature phenotype, with low expression levels of KIRs and CD16, as well as higher expression level of NKG2A. Genetically engineering iNK cells to express non-cleavable CD16 Fc receptor to mediate the ADCC effect may be a reasonable solution to this limitation (58, 59). FT596 and FT576, cell products for B-cell malignancies and MM, respectively, are investigational and off-the-shelf CAR-NK cell products derived from a human clonal master iPSC line engineered with anti-tumor abilities. These functional cell products can be manufactured on a large scale to support multi-dose therapeutic strategies and on-demand dose availability (60–62).

### Transduction of CAR-Gene Into NK Cells

Currently, low transduction efficiency caused by the lack of efficient gene transferring approaches is a major barrier. The methods used for transduction of CARs into T cells are also applicable in NK cells. Approaches include viral transduction (retrovirus-based and lentivirus-based) and transfection (electroporation, lipofection, and in combination with transposon systems) (63). Viral-based transduction approaches enable stable integration into the genomes of CAR-NK cells. Retroviral vector transduction exhibits high efficacy (43–93% in primary NK cells), while the insertional mutagenesis and deleterious impact represent major restrictions of this approach in clinical applications (64). Nevertheless, lentivirus-based transduction is safer, while its transduction efficiency (8–16% in PBMC-derived NK cells) must be further improved (65). RNA transfection methods are cost-effective approaches with higher gene transfer efficiency; however, the CAR constructs expression is transient through this method, approximately 3–5 days. On the one hand, the narrow therapeutic time window is a limitation; on the other hand, the incidence of CAR-associated side effects, such as on-target off-tumor effects, may be reduced due to its transient nature (39, 66, 67). Combining transfection methods with DNA integration techniques through transposon systems, such as PiggyBac (PB) and sleeping beauty (SB), has been developed as an attractive approach to generate safer and more stable transgene-expressing cells (68, 69). The SB transposon vector has provided an efficient and economic method for gene transfer, while its applicability in CAR-NK cells remains untested (70).

## Applications of CAR-NK Cells in Hematological Malignancies

### B-Cell Lymphoma and Leukemia

B-cell originated lymphoma and leukemia which belong to hematological B-cell malignancies, are characterized by the expression of one or more common B-cell antigens (71). CAR-T-based cellular therapy is thriving and promising in the management of B-cell malignancies. Frequently used target antigens in CAR-T cell therapy for B-cell lymphoma and leukemia (e.g., CD19, CD20, and CD22) are also applicable in CAR-NK cells (72, 73). Multiple preclinical and clinical trials have been conducted to test the efficacies of CAR-NK cells for B-cell malignancies (**Table 1**).

**Table 1:** Clinical trials of CAR-NK cells in hematological malignancies.

No.	Target	Conditions	NK cell sources	Status	Phase	NCT
1	CD19	B-cell malignancies	UCB	recruiting	phase I	NCT04796675
2	CD19	B-cell NHL	unknown	not yet recruiting	early phase I	NCT04639739
3	CD19	B-cell lymphoma	iPSCs (FT596)	recruiting	phase I	NCT04555811
4	CD19	B-cell malignancies	iPSCs (FT596)	recruiting	Phase I	NCT04245722
5	CD19	B-cell NHL	iPSCs	not yet recruiting	early phase 1	NCT03824951
6	CD19	B-Cell lymphoma	unknown	not yet recruiting	early phase I	NCT03690310
7	CD19	B-cell malignancies	UCB	completed	phase I/II	NCT03056339 ref (31).
8	CD19	B-cell malignancies	NK-92 cells	unknown	phase I/II	NCT02892695
9	CD19	B-cell ALL	PBMCs	completed	phase I	NCT00995137
10	CD22	Refractory B-Cell Lymphoma	unknown	not yet recruiting	early phase I	NCT03692767
11	CD19/CD22	Refractory B-Cell Lymphoma	unknown	not yet recruiting	early phase I	NCT03824964
12	CD7	T-cell malignancies, AML	NK-92 cells	recruiting	phase I/II	NCT02742727
13	CD33	AML	NK-92 cells	completed	phase I/II	NCT02944162 ref (74).
14	NKG2D Ligands	AML, MDS	PBMC	recruiting	phase I	NCT04623944
15	BCMA	MM	NK-92 cells	recruiting	phase I/II	NCT03940833

Engineering NK92 cells with an anti-CD19 CAR construct that incorporates a CD3ζ signaling domain either alone or with additional costimulatory molecules (e.g., CD28, 4-1BB, or 2B4) could achieve significant cytotoxicity of NK cells towards B-cell leukemic cell lines and NK-resistant primary leukemia cells *in vitro* (64, 75–77). These studies have shown that the integration of either 2B4 or CD28 could further enhance all aspects of the CD19 CAR-NK cell activation response to B-cell leukemia cells (76, 77). Moreover, concerning the potential risk of malignant transformation through transfusion of NK92 cells, Liu et al. provided evidence in mouse models that irradiation before infusion of CD19 CAR-NK92 cells could avoid the potential risk of secondary NK lymphoma without the reduction of killing capacity against CD19+ malignancies, compared with unirradiated cells (78). The therapeutic potential and effectiveness of CD20 CAR-NK cells for CD20+ aggressive B-cell non-Hodgkin’s lymphoma (NHL) have also been confirmed in mouse models by Chu et al. (79, 80). Another group designed dual CD19/CD22 CAR-NK cells, which can kill both CD19^KO^- and CD22^KO^-RS4;11 cells (an ALL cell line) *in vitro* (81).

Clinical trials registered on http://www.clinicaltrials.gov involving CAR-NK cell therapy in hematological malignancies are summarized in **Table 1**. CD19 is a popular target in both CAR-T and CAR-NK cell therapy methods; many clinical trials are focused on B-cell malignancies. Thus far, only one Phase I/II large-scale clinical trial has been published (31). Eleven heavily pretreated patients with CD19+ relapsed or refractory (r/r) chronic lymphocytic leukemia (CLL) or NHL were enrolled in the clinical trial; they received UCB-derived CAR-NK cells with a novel construct incorporating the IL-15 gene and an inducible caspase-9-based suicide gene after lymphodepleting chemotherapy. One of three doses (1×10^5^, 1×10^6^, or 1×10^7^ CAR-NK cells per kilogram of body weight) of CAR-NK cells was infused into different patients; a maximum tolerated dose was not reached. Responses were evident within 30 days: eight of eleven patients had a response, seven (four with lymphoma and three with CLL) of whom exhibited complete remission. With the inclusion of the IL-15 construct, the infused CAR-NK cells persisted at least 12 months *in vivo*, as measured by a quantitative real-time polymerase-chain-reaction assay. After the infusion of CAR-NK cells, all the patients suffered transient and reversible hematologic toxic events, such as neutropenia, lymphopenia, lymphopenia and anemia, which were mainly caused by the lymphodepleting chemotherapy. However, there were no cases of CRS, neurotoxicity, hemophagocytic lympho-histiocytosis, tumor lysis syndrome or grade 3 or 4 nonhematologic toxicity. Despite the HLA mismatch between patients and their CAR-NK products, no symptoms of GVHD were observed. As a result, the caspase 9 safety switch was not activated because of the absence of severe adverse events in the clinical trial. FT596, a universal off-the-shelf CAR-iNK cell product that contains CD19, hnCD16, and an IL-15 receptor, is currently under investigation in two clinical trials (NCT04555811 and NCT04245722). The aim of the Phase I multi-center study (NCT04555811) is to evaluate the safety of FT596 when given with rituximab as relapse prevention in patients who have undergone auto-HSCT for B-cell lymphoma. In the past 6 months, two new clinical trials (NCT04796675 and NCT04639739) have been registered concerning CD19 CAR-NK cells, indicating that the promising clinical trial results have aroused the interest of researchers in the field of CAR-NK cell immunotherapy and bringing new hope for patients with B-cell malignancies. In addition to CD19 CAR-NK cells, the anti-tumor activation and safety of CD22 CAR-NK cells for B-cell malignancies are also under investigation in clinical trials (NCT03692767 and NCT03824964).

### Multiple Myeloma

MM, comprising approximately 10% of hematological malignancies, is a clonal plasma cell disorder that produces excess monoclonal immunoglobulin. The disease often features hypercalcemia, renal failure, anemia, and bone lesions (82).

B-cell maturation antigen (BCMA) is widely expressed on plasma cells, myeloma cell lines, and primary myeloma while absent in primary human CD34+ hematopoietic cells (83). CAR-T cells that target BCMA have been mostly used in MM; CD38, CD138, and CD319 (CS1) are also common target antigens for MM (84). Additionally, the NKG2D ligand (overexpressed on MM cells) is a potential target for MM. Because CAR-T-cell therapy has generated considerable enthusiasm in patients with MM, CAR-NK cell therapy for MM is also a focus of research.

FT576 NK cells, a CAR-NK cell type derived from iNK cells, exhibit uniform expression of CD16, anti-BCMA CAR, and IL15-receptor α fusion protein (IL-15RF) and did not express CD38 (62). Preclinical trials proved FT576 NK cells enhanced cytotoxicity and persistence, avoidance of self-fratricide, and prevention of antigen loss when combined with other mAbs (e.g., anti-CD38 mAb) in the treatment of MM (61, 62). Chu et al. indicated that CS-1 CAR-NK92 cells displayed CS1-dependent recognition and killing capacity for both MM cell lines and primary MM cells; this cell therapy prolonged survival in xenograft model mice by inhibiting the growth of MM cells (85). Moreover, NK cells carrying a CD138 CAR exhibited enhanced cytotoxicity against CD138+ human MM cell lines and primary MM cells with increased secretion of granzyme B and IFN‐γ, as well as increased expression of CD107a, both *in vitro* and in xenograft models (86).

A clinical trial (NCT03940833), conducted by Wuxi People’s Hospital, plans to recruit 20 patients with MM. The purpose of that study is to assess the safety and feasibility of BMCA CAR-NK92 cells for patients with r/r MM.

### T-Cell Lymphoma and Leukemia

T-cell malignancies mainly include T-lymphoblastic leukemia and T-lymphoblastic lymphoma, and T-lymphoblastic lymphoma can be further divided into two categories: cutaneous T-cell lymphoma (CTCL) and peripheral T-cell lymphoma (PTCL). Although CAR-T cell therapy has been successfully used in B-cell malignancies, it generally has not shown good results in the treatment of T-cell malignancies because of the shared expression of target antigens between normal and malignant T cells, which may lead to fratricide and immunodeficiency (87). Thus far, molecules used as the targets for CAR-based cellular therapy in T-cell malignancies are CD3, CD4, CD5, CD7, CD30 and CD1a (88–93). Recently, preclinical and clinical trials have been initiated using CAR-NK cells as a therapy for T-cell lymphoma/leukemia.

Chen et al. have generated CD3 CAR-NK92 cells and demonstrated specific and effective lysis of CD3+ human PTCL primary samples, as well as T-cell leukemia cell lines. Moreover, CD3 CAR-NK92 cells controlled the growth of T-ALL in xenogeneic mouse models, with an 87% tumor burden reduction on day 13 and maintenance at this level on day 23 (94). Furthermore, the same group has confirmed the safety and effectiveness of CD4 CAR-NK92 cells against various CD4+ T-cell lymphoma/leukemia cell lines and patient samples (95). Wang et al. compared two CD5 CAR-NK cells with different costimulatory molecules: 4-1BB and 2B4. Their findings suggested that NK-cell-associated activating receptor 2B4 may further enhance the killing activity of CD5 CAR-NK cells against CD5+ malignancies, compared with T-cell-associated activating receptor 4-1BB (96, 97). The median survival time of xenogeneic mice was longer in the CD5-2B4-CAR-NK92 group than in the CD5-4-1BB-CAR-NK92 group: 45.5 days and 58.5 days, respectively (p < 0.05) (97). You et al. first reported two CD7 CAR-NK92-MI cell constructs using the CD7 nanobody VHH6 sequences; they demonstrated that CD7 CAR-NK92-MI cells exert specific cytotoxicity and an inhibitory effect on primary T-ALL cells in a PDX mouse model (98).

Currently, only one clinical trial (NCT02742727) related to T-cell malignancies is registered; the purpose of this trial is to evaluate the safety and effectiveness of CD7 CAR-NK92 cell immunotherapy in patients with CD7+ r/r lymphoma and leukemia. However, this study has not yet been published. The shared antigens between tumor cells and healthy tissues remain a major challenge for CAR-based cellular immunotherapy in T-cell malignancies. Thus, there is a need for approaches to seek out new target antigens expressed exclusively on tumor cells or identify the appropriate therapeutic windows of CAR-NK cells to minimize T-cell depletion (96).

### Myeloid Malignancies (AML/MDS)

Myelodysplastic syndromes (MDS) and acute myelogenous leukemia (AML) are two mainly myeloid malignancies, characterized by uncontrolled proliferation of undifferentiated myeloid progenitor cell clones (99). With the progression of disease, there is a risk of MDS conversion to AML. Application of CAR-T cell therapy in AML is difficult because nearly all currently known target antigens are expressed both on leukemic blasts and healthy progenitor cells. Therefore, methods using CAR-T cells as salvage therapy or as a bridge to allo-HSCT for AML may circumvent these challenges (100). Potential target antigens (e.g., CD33, Lewis Y, CD123, CD135, CLL1, CD44v6, FRβ, CD38, and CD7) for AML have been summarized by Hofmann et al. (101). Klöß et al. generated CD123 CAR-NK cells and showed excellent capacity for eliminating resistant AML blasts and AML cell lines ex vivo (102). In addition to expression in T-cell malignancies, CD4 antigen is also expressed in a subtype of AML; therefore, CD4 CAR-NK cells also exhibit potent antileukemic activity against CD4+ AML (103). A first-in-man small clinical trial (NCT02944162) of CD33 CAR-NK92 cells for patients with r/r AML has been completed with published data (74). In this clinical trial, each of three patients received doses of up to 5 × 10^9^ CD33 CAR-NK92 cells. Among them, two patients suffered a moderate fever and one patient suffered a high fever after CD33 CAR-NK92 cell infusion, and all of them returned to normal within in two days. Grade I CRS was observed in patient 1 and 3. No other significant adverse effects were observed. Though no conclusion regarding clinical efficacy could be drawn, the safety of using CD33 CAR-NK92 cells for r/r AML patients with high tumor burden was clear.

In addition to the target antigens mentioned above, NKG2D ligands are promising target antigens because of their increased expression in AML and MM, but absence in healthy tissues (104). The safety and capacity of manufactured CAR-T cells that target NKG2D ligands for AML patients and MM patients have been verified in a phase I clinical trial (105). Recently, one new clinical trial (NCT04623944) was registered to determine the safety and tolerability of an experimental therapy known as NKX101 (allogeneic CAR-NK cells targeting NKG2D ligands) in patients with r/r AML, r/r MDS or intermediate (high/very high)-risk MDS.

Currently, similar to CAR-T cell therapy, clinical trials about CAR-NK cell therapy are mainly focused on r/r patients only. Several studies have suggested that the infusion of unmodified NK cells is feasible to maintain complete remission (CR) status for MDS or AML patients (55, 106, 107). Besides, adoptive NK cell transfer either alone or combined with chemotherapy could also be used as a post-CR consolidation strategy in AML (108, 109). A phase II trial that transfusing NK cells as a consolidation therapy in pediatric patients with AML has been conducted, and the result is still awaited (110). Additionally, one study also suggested that infusing NK cells could also consolidate incomplete engraftment in patients after haploidentical HSCT (19). Therefore, it is reasonable to assume that in addition to r/r patients, CAR-NK cells may also hold the potential to be used in consolidation and maintenance stages for treating myeloid malignancies. We believe that the potential clinical applications of CAR-NK cell therapy will be further extended in the future.

## Advantages of CAR-NK Cell Therapy, Compared With CAR-T Cell Therapy

The comparisons between CAR-T cells and CAR-NK cells are summarized in **Table 2**. CAR-NK cells possess several strengths because of the unique biological features compared with CAR-T cells. Here, we illustrate the advantages of CAR-NK cells in immunotherapy.

**Table 2:** Comparisons between CAR-T cells and CAR-NK cells.

	CAR-T cells	CAR-NK cells
Sources	mostly autologous T cells; using allogenic T cells may cause GVHD	various sources: PBMCs, UCB, NK cell line, iPSCs, hESCs, HPCs
Transduction efficiency	higher	lower
*In-vivo* persistence	better	worse
Safety	using allogeneic T cells may cause GVHD;	rarely cause GVHD, may even protect against GVHD;
	CRS and neurotoxicity are two acute side effects observed in CAR-T cell immunotherapy	no CRS and neurotoxicity observed in CAR-NK cell immunotherapy
Efficacy	high: recognize tumor cells through the CAR-dependent manner	higher: recognize tumor cells through both CAR-dependent
		and CAR-independent manners
Convenience	less convenience: necessities of matching HLA;	more convenience: the possibilities to be an off-to-shelf
	the consuming manufacturing time and expensive price	products because of no HLA-restriction and various sources
Current status	two CD19 CAR-T cells have been approved by the FDA;	preclinical and clinical trials have been conducted;
	new types of CAR-T cells have been conducted in clinical trial	several published data are available now

Firstly, CAR-NK cells have superior safety than CAR-T cells. There are two main reasons for the superior safety of CAR-NK cells. First, CRS and neurotoxicity are two common adverse effects observed in CAR-T cell therapy. Abundant cytokines are released in the circulation and tissues, leading to various levels of symptoms, such as high fever, sinus tachycardia, hypotension, hypoxia, depressed cardiac function, and other organ dysfunction (12). The cytokine storm induced by CAR-T cells is mainly mediated by pro-inflammatory cytokines (e.g., TNFα, IL-1, and IL-6) (111). While CAR-NK cells secret a spectrum of cytokines (e.g., IFN-γ and GM-CSF) that are different from those secreted by CAR-T cells. Second, CAR-T cells, either from autologous or allogeneic sources, may cause life-threatening GVHD due to HLA restriction, due to HLA restriction. Conversely, NK cells, regarded as major effector cells that mediate early GVL reaction, may prevent GVHD by killing recipient antigen-presenting cells (APCs) and cytotoxic T lymphocytes (112). Therefore, the use of CAR-NK cells could eliminate safety concerns regarding clinical applications, compared with CAR-T cell products.

Secondly, CAR-NK cells may have better efficacy in attacking tumor cells than that of CAR-T cells. First, CAR-NK cells can recognize and exert their killing effect through engineered killing capacity and intrinsic killing capacity (**Figure 1**). Through CARs, effector cells can focus their killing capacity on a particular antigen in a more efficient manner. Unlike CAR-T cells, CAR-NK cells still preserve the natural cytotoxicity of NK cells in case of downregulated expression of targeted tumor antigens (113). NK cells recognize their target cells and then play biological roles involving various mechanisms: (1) natural cytotoxicity; (2) ADCC effect; (3) TNF-related apoptosis-inducing ligand (TRAIL); and (4) FAS/FASL (114). Of note, NK cells maintain dynamic balance and intricate interactions through various activating and inhibitory receptors. After NK cells have been activated, cytotoxic granules (e.g., granzyme and perforin) are released by NK cells; these powerful weapons promote the apoptosis of target cells. Second, in addition to the costimulatory domains shared with CAR-T cells (e.g., CD28 and 4-1BB), CAR-NK cells exhibit specialized molecules with greater costimulatory specificity in NK-cell signaling (e.g., DAP10, DAP12, and 2B4), as mentioned in the section *CAR Constructs*. Preclinical data have indicated enhanced cytotoxic killing capacity of CAR-NK cells engineered with these costimulatory molecules (39, 77, 97).

Thirdly, the process of manufacturing CAR-NK cells is more convenient than the process of manufacturing CAR-T cells. Because the risk of GVHD is absent, NK cells can be isolated from either matched or HLA-mismatched donors, thus providing more choices of possible donors and increasing the quality of the final products (115). Various sources of NK cells (e.g., NK92 cell lines, PBMC-derived NK cells, UCB-derived NK cells, and iPSC-derived NK cells) have been used to generate CAR-NK cells. Thus, the low possibility of GVHD and various sources of NK cells may enable NK cells to serve as “off-the-shelf” products which could readily be available for clinical use.

## Challenges and Prospects of CAR-NK Cells

Although there are many advantages of CAR-NK cells in cancer immunotherapy, compared with CAR-T cells, challenges remain to influence the function and efficacy of CAR-NK cells. In contrast to T cells and other human cells, NK cells are more sensitive to the process of freezing and thawing, reducing their anti-tumor capacity and survival rate. These limitations may restrict the ability to distribute CAR-NK cells to distant places in an “on-demand” manner (49, 116); Furthermore, limited expansion and persistence capacity is a major problem when using NK cells and their engineered products as adoptive immunotherapy (49). Immunosuppressive cytokines (e.g., TGFβ, adenosine, and indoleamine 2,3-dioxygenase), secreted in the immuno- suppressive tumor microenvironment (TME), have negative effects on CAR-NK cells (117). Inhibitory receptors (e.g., immune checkpoint molecules [TIGIT, PD-1, and CTLA-1], C-type lectin receptor [NKG2A] and cytokine checkpoint [CISH]) also contribute to CAR-NK cell dysfunction (118). Therefore, future considerations and prospects should be put forward to maximize the efficacy of CAR-NK cell immunotherapy.

First, improving CAR-NK cell constructs to overcome the limitations and fully unleash the potential of CAR-NK cells is critical in CAR-NK cell immunotherapy. Cytokines (e.g., IL-2, IL-12, and IL-15) are critical for enhancing the activity, persistence, and expansion of NK cells in both innate and adaptive immunotherapy (119). Moreover, the capacity of frozen NK cells could be partially recovered by the addition of IL-2 (120). Therefore, transducing cytokine genes and knocking out inhibitory genes through gene modification in CAR-NK cells are promising methods. Because of the potential for unanticipated toxicity due to excessive cytokine production by novel CAR-NK cells, the incorporation of a suicide gene into CAR-NK cells is an important consideration for safety concerns. Daher et al. have shown that C9/CAR.19/IL-15 CB-NK cells can be readily eliminated upon pharmacologic activation of the iC9 suicide gene in both preclinical and clinical studies (31, 121). They also showed that the deletion of CISH, a gene encoding a cytokine checkpoint molecule, enhances the metabolic fitness and antitumor activity of armored IL-15-secreting CD19 UCB-derived CAR-NK cells in lymphoma models (122). NK cells engineered to express CARs and other exogenous genes, which are called “armored” CAR-NK cells or “NK-cell pharmacies”, can exhibit multiple functions (123).

Second, discovering and selecting appropriate TAAs expressed on tumor cells exclusively is essential to avoid on-target off-tumor effects in CAR-based immunotherapy. Target antigens in T-cell malignancies and myeloid tumors are often expressed on both tumor blasts and healthy tissues, which leads to severe toxicity (124). In recent years, NKG2D ligands have emerged as new potential targets. The safety of NKG2D CAR-NK cells in treating MDS/AML and MM has been confirmed (105). New clinical trials are underway for detailed information concerning the novel engineered cells.

Because of the unique natural cytotoxicity of NK cells, CAR-NK cells could target and kill tumor cells in a CAR-independent manner. Therefore, designing a non-signaling CAR structure that focuses on homing-promoting target factors (e.g., chemokines and adhesion molecules), rather than target antigens that induce a direct killing signal, is a potential solution. The novel CARs allow CAR-NK cells to gather into tumor sites; thus, CAR-NK cells could function in a NK cell-mediated manner, rather than a CAR-dependent manner (63, 125). In hematological malignancies, this may be more suitable in lymphoma because of the specific tumor site. Hence, normal tissues and cells could be protected from on-target off-tumor toxic effects because of the “missing-self” mechanism of NK cells (126).

Third, cytokines, immune checkpoint inhibitors, and monoclonal antibodies could enhance the cytotoxicity of NK cells in adoptive immunotherapy, as summarized above. Similarly, transfusion of CAR-NK cells combined with the above approaches could further enhance the killing capacity and safety of CAR-NK cells.

Chemotherapy and radiation therapy are widely used as adjuvant therapeutic approaches in cancer therapy. Administration of lymphodepleting chemotherapy and radiation before the infusion of CAR-NK cells could reduce the tumor burden and increase the effector-to-target ratio after transfusion of CAR-NK cells (46).Additionally, radiotherapy-related DNA damage may induce the expression of NKG2D ligands on tumor cells and enhance the cytotoxicity of CAR-NK cells against tumor cells (127).

Several clinical trials have confirmed that CAR-T cell therapy is a safe and effective therapeutic strategy for r/r B-ALL patients when used as a bridging strategy before HSCT (128). Thus, CAR-NK cells may also serve as a bridge therapy, through which patients can achieve a low pre-infusion minimal residual disease (MRD) status before administration of allo-HSCT. The purpose of an ongoing clinical trial((NCT02892695) is to evaluate the safety and optimal dose of CD19 CAR-NK cells used as a bridge therapy in patients who plan to receive HSCT.

## Conclusions

The emergence of CAR-T cells is a breakthrough in cancer immunotherapy, especially for B-cell malignancies. However, due to the risk of severe adverse effects, such as CRS, neurotoxicity, and GVHD, the research focus has been shifted from CAR-T cells to CAR-NK cells. Preclinical studies and clinical trials have been conducted to confirm the safety and efficacy of CAR-NK cell therapy in treating hematological malignancies. Thus far, only two clinical trials were published with available data. The results of ongoing clinical trials are still eagerly awaited. NK cells, as new effector cells used in CAR-based immunotherapy, exhibit their unique advantages. Different from CAR-T cells, CAR-NK cells could attack tumors through both CAR-dependent and CAR-independent manners. The abundant sources and the lack of HLA-matching restriction of NK cells enable the generation of “off-the-shelf” CAR-NK cell products that could be manufactured in advance and prepared for clinical use. Besides, CAR-NK cells are safer than CAR-T cells because of the low incidence of adverse events. However, there are also challenges influencing the function and the efficacy of CAR-NK cells. NK cells are sensitive to the process of freezing and thawing, which will reduce their anti-tumor capacity and survival rate. Besides, the limited expansion and persistence capacity is also a restriction for CAR-NK cells. Many approaches are still under investigation to further minimize the adverse effects and improve the function of CAR-NK cells. Currently, the clinical trials of CAR-NK cell therapy are mainly focused on r/r patients. Therefore, the potential applications of CAR-NK cells are also need to be explored.

## Author Contributions

HW, YH, and HL designed this work. HL, XZ, and ZL wrote this manuscript. All authors contributed to the article and approved the submitted version.

## Funding

The study was supported by grants from the National Natural Science Foundation of China, No.81770134.

## Conflict of Interest

The authors declare that the research was conducted in the absence of any commercial or financial relationships that could be construed as a potential conflict of interest.

## Publisher’s Note

All claims expressed in this article are solely those of the authors and do not necessarily represent those of their affiliated organizations, or those of the publisher, the editors and the reviewers. Any product that may be evaluated in this article, or claim that may be made by its manufacturer, is not guaranteed or endorsed by the publisher.
